# Use of a dark blood sequence to localize the esophagus prior to RF ablation and to assess left atrial edema post ablation

**DOI:** 10.1186/1532-429X-11-S1-P214

**Published:** 2009-01-28

**Authors:** Sathya Vijayakumar, Eugene G Kholmovski, Edward VR DiBella, Josh R Bertola, Nassir F Marrouche

**Affiliations:** University of Utah, Salt Lake City, UT USA

**Keywords:** Left Atrial, Pulmonary Vein Isolation, Electron Beam Tomography, Left Atrial Wall, Pulmonary Vein Ostium

## Aim

The aim of this study is to present an MRI technique to localize the esophagus prior to RF ablation procedure, while also tracking left atrial (LA) wall thickness as a sign of acute edema post-ablation.

## Introduction

Lately, ablation targeting arrhythmogenic pulmonary vein (PV) foci for drug-refractory paroxysmal atrial fibrillation has become one of the standard methods of pulmonary vein isolation (PVI). CT or MRI is commonly performed prior to ablation for procedure guidance. Localization of the esophagus is important pre-ablation to prevent fatal atrio-esophageal fistula, especially in view of a need for transmural atrial lesions. While CT is relatively established for esophageal localization [1, 2], MRI may also be useful for this purpose. Also, measuring LA edema after PVI could increase understanding of patient response to the procedure. Okada et al [3] recently reported the use of electron beam tomography to visualize LA edema after PVI. Here, we present the use of a dark blood MRI sequence that can localize the esophagus and be used to measure LA wall thickening, indicative of edema.

## Methods

This study obtained and assessed data from 13 patients aged 68.8 ± 11.1 years ranging from 49–83 years of age, 7 male and 6 female. Patients were imaged before PVI procedure, to help localize the esophagus, 24 hours, and 3 months post-procedure to assess the presence of LA edema. A respiratory navigated, black blood prepared turbo spin echo sequence was used with a TR of 1 RR, TE 61 ms, ETL 22, slice thickness 4 mm, FOV 36 mm, 288 × 288 matrix, parallel imaging with reduction factor 2 and 44 reference lines. 18–22 continuous transaxial slices were acquired to cover the LA.

**Figure 1:**
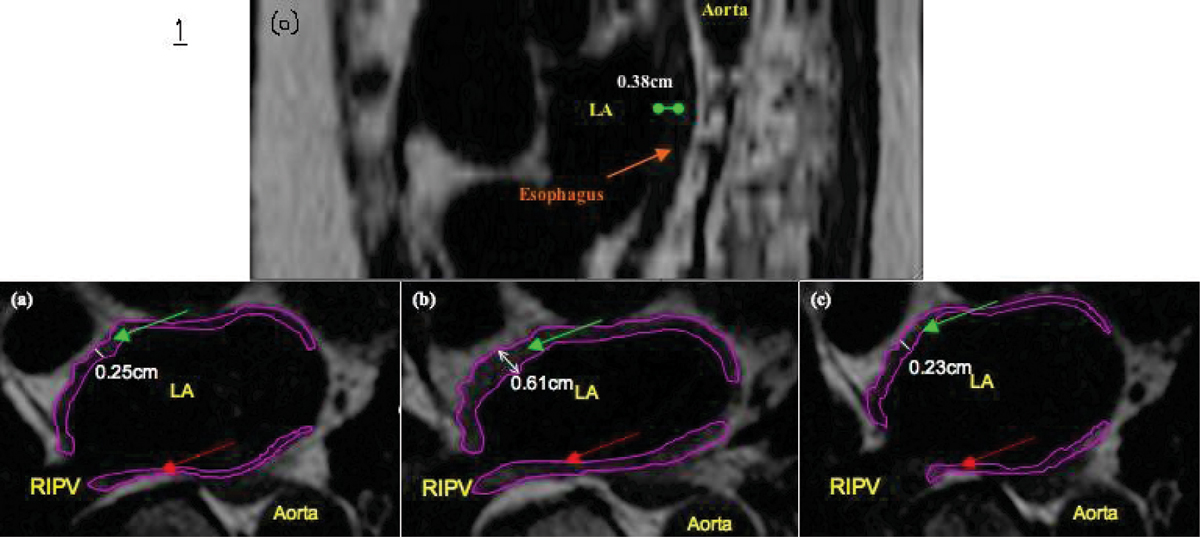
Figure 1

Image analysis was performed using the OsiriX Imaging Software. For localization of the esophagus with respect to PVs, the distance between the center of each PV ostium and the center of the esophagus was measured. The distance between the center of esophagus and posterior wall of the LA was assessed in a similar way (Figure 1(o)).

Figures 1(a,b,c) illustrate an example of how wall thickness was assessed, and how contours were drawn for measurement of areas of the anterior and posterior wall of the LA. Paired Student's t-test was used to compare the width of the LA wall, and areas of the anterior and posterior walls of the LA, at the three timepoints (pre, 24-hour and 3-month post procedure).

## Results

Table 1 shows distances between the esophagus and PV ostia and posterior wall of LA. The mean % increase in wall thickness and areas of posterior and anterior walls of the LA, measured 24-hours post ablation, were 53 ± 22, 51 ± 29 and 42.6 ± 16 respectively. P < 0.0001 for all three of them implies this statistically significant increase in LA wall thickness is likely from acute edema of the LA wall [3]. It was also determined that the wall thickness, areas of posterior and anterior walls measured pre-ablation and 3-month post-ablation were not statistically significantly different (p = 0.095, p = 0.51, p = 0.24) respectively, implying that the edema visualized 24-hour post PVI procedure was resolved by 3 months post-procedure.

**Table 1:** Mean ± SD for relative distances of the esophagus from the LIPV, RIPV and posterior wall of LA

Distance from LIPV (cm)	Distance from RIPV (cm)	Distance from posterior wall of LA (cm)
2.02 ± 1.04	3.64 ± 0.89	0.39 ± 0.06

## Conclusion

This study shows that using MRI, the esophagus can be localized prior to ablation, and edema caused as a result of PVI using RF ablation can be successfully visualized. LA wall thickness changed significantly between pre and 24-hour post procedure images. With time, this edema was found to resolve itself and the measurements made 3 months post-procedure and pre-procedure were not statistically significantly different. The results obtained are comparable to those recently reported using CT.
